# Iodoform with Iron as an Alterative

**Published:** 1876-10

**Authors:** A. L. Reichard

**Affiliations:** Allentown, Pa.


### IODOFORM WITH IRON AS AN ALTERATIVE.

By A. L. REICHARD, M.D., Allentown, Pa.

Writing communications for papers or medical journals has never been my forte, but on looking over my note book, I find recorded cases which were treated with that invaluable remedy, pills of iodoform and iron, and, after having used them for a period of nine years, I am free to say that in chronic ulceration, I care not to what extent it may exist, or of how long standing it may be, it will cure them; not for a short period only, and then break out afresh, an occurrence that I have never witnessed in my experience. I have treated some of the worst forms of chronic ulcer, and I have the first case to see where the cure was not permanent and lasting, and in this I simply corroborate the experience of Dr. Stiles Kennedy, published in 1870. One case in particular that I treated in 1867, nor can I conceive the possibility of a worse case, of which I made a full report to, and which was published by, the State Medical Association in its annual transactions, as the report of the Lehigh County Medical Society in 1868.

The following is a brief recapitulation of the extent of ulceration and the final treatment:

Female, age thirty, ulceration of six years' standing, had been under medical treatment nearly that length of time before she came to this city. Her inferior extremity was affected from the knee to and involving the ankle joint.

After I had exhausted all the usual and well as unusual remedies for several months, I found she had not advanced a single step toward improvement. I then discontinued all other treatment and put her on pills of iodoform and iron, made by Wm. R. Warner & Co., of Philadelphia, three *ter die*, when the change was like magic, and in an incredibly short time she was discharged *perfectly cured.*

I merely recall the case to show that the cure was perfect and permanent, for she remained in a healthy condition until the day of her death, which was recent, (of typhoid), nine years from the time I discharged her. Not in the slightest degree did the disease reappear. This will assure you that the remedy will do, and what may be expected of it. If properly used it cannot fail· I have not only used it in the above case, but in neuralgia, in all its forms, I have had the same gratifying results.

In the winter of 1874 I had a lady under treatment, who had suffered for eighteen years with what she called "womb disease."

On making an examination, I found hypertrophy of the cervix uteri, but no ulceration, though she was suffering most agonizing neuralgic pain.

She told me that she had doctored so much since her first confinement (now eighteen years) that she despaired of ever being cured. She would be confined to her bed for weeks at a time, and when she did obtain relief it was of short duration. She never was perfectly free from pain for one continuous month during those eighteen years.

I took charge of the case, and of course told her that I could relieve her, (and with some degree of confidence), as all others had told her. But after treating her for some months, I signally failed, and was obliged to fall back in line with those who had done battle before me. I was repulsed, but not conquered.

I gathered up a new weapon of defence in the form of the remedy above named, iodoform and iron, and attacked the enemy again, by giving her two pills *ter die*, and in two weeks she was down stairs, free from pain. I continued this treatment until she had taken two hundred; since then she has not had a single pain (now sixteen months.) To use her own language, "She is a new being." The hypertrophy has disappeared, and she is in better health than she has been for years.

During the period of her suffering she was deprived of many of the comforts and pleasures of life; riding was torture, now t is one of her chief pleasures, as she can visit her friends, and has gone to New York and returned with perfect comfort, a thing which was not at all thought of prior.

In conversaiion with Dr. E. G. Martin, of this city, he related several cases of this character which he has treated with almost immediate success *with this remedy*. One case in particular, a very obstinate one, that had resisted all treatment. She would suffer most intense pain notwithstanding every possible means was brought to bear upon her case, including the galvanic battery but all with no effect. If relief was obtained it was soon followed by another paroxysm of pain, until he commenced the odoform pill treatment, when every vestige of pain left her, and thus far she has not had a return of the trouble (now two years.)

My friend, F. C. Seiberling, gave me a history of a very interesting case, some time since, which goes very far to prove what has been already written on the treatment of sciatica with pills of iodoform. The doctor had a very severe and obstinate case of sciatica, which stood out against all treatment and remedies indicated in this affection. After he had exhausted his patience, as well as that of his patient, the case fell into the hands of another physician, who treated him a long time with the same discouraging result, when the patient again applied to my friend Dr. Seiberling, who at once put him upon the iodoform pill treatment, and, strange to say, after twelve pills had been taken the pain ceased, the disease was *completely routed.* He continued treatment for a while to insure a radical cure.

I could go on citing case after case of cure by this remarkable remedy, until I would tire your patience in the perusal, and then not be at the end. But let this suffice for the present.—*St. Louis Clin. Rec.*

